# Audiovestibular clinician experiences and opinions about cisplatin vestibulotoxicity

**DOI:** 10.1007/s00405-020-06033-4

**Published:** 2020-05-19

**Authors:** Pattarawadee Prayuenyong, Anand V. Kasbekar, Deborah A. Hall, David M. Baguley

**Affiliations:** 1 Hearing Sciences, Division of Clinical Neuroscience, School of Medicine, University of Nottingham, Nottingham, UK; 2 NIHR Nottingham Biomedical Research Centre, Ropewalk House, 113 The Ropewalk, Nottingham, UK; 3 Nottingham University Hospitals NHS Trust, Nottingham, UK; 4 University of Nottingham Malaysia, Semenyih, Malaysia; 5 Department of Otorhinolaryngology, Head and Neck Surgery, Faculty of Medicine, Prince of Songkla University, Songkhla, Thailand

**Keywords:** Audiovestibular, Cisplatin, Vestibular, Vestibulotoxicity

## Abstract

**Purpose:**

Vestibulotoxicity associated with cisplatin chemotherapy is known to exist, but the extent, severity, and impact is unclear from the literature. This study explored knowledge, experiences, and opinions of audiovestibular professionals about cisplatin vestibulotoxicity.

**Methods:**

An online survey was disseminated to clinicians working in the audiovestibular field.

**Results:**

Ninety-three respondents participated in the survey. Most professionals were aware of potential vestibulotoxicity associated with cisplatin chemotherapy. Thirty-three percent of the respondents reported that they had seen patients with cisplatin vestibulotoxicity. Forty percent of them were confident in making the diagnosis and in managing the patient in this situation. The prevalence and impact of vestibulotoxicity including practicality of the assessment should be considered when designing an effective vestibulotoxicity screening protocol.

**Conclusion:**

This study provides a better understanding of cisplatin vestibulotoxicity from the perspectives of audiovestibular clinicians, which will underpin appropriate detection and management of the condition.

## Introduction

Cisplatin is a highly effective chemotherapeutic agent against a variety of life-threatening cancers, but its ototoxic effect is considerably problematic and limits usage and dosage [1]. Ototoxicity refers to drug-related damage affecting the inner ear structures, which can be characterized by cochlear dysfunction or vestibular dysfunction or both [2]. A variable degree of irreversible hearing loss as a result of cisplatin treatment is well documented with a reported prevalence of 50–90%, depending on patient demographics, drug dosage, and differences in tools and grading system [3–5].

Given that the auditory and vestibular organs of the inner ear share vascular, neural, and fluid supplies [6, 7], an ototoxic drug may affect both compartments. The vestibular part of the inner ear plays a vital role in the complex and dynamic human balance system, together with interactions of visual, somatosensory, and central nervous systems [8]. Balance problems such as dizziness and unsteadiness can cause significant negative impact on quality of life [9] and substantial economic burden [10], especially in a vulnerable group of cancer survivors [11]. Whilst the existence of cisplatin vestibulotoxicity is evident [12], the extent, severity, and impact are largely unclear from the literature, and seem to be under-reported and under-investigated.

Hearing surveillance to monitor cochleotoxic effects associated with cisplatin chemotherapy is advised and implemented in clinical practice [13, 14]. On the other hand, we are unaware of any established protocol for monitoring vestibular function, and for the detection of balance dysfunction following exposure to potential ototoxic medications. This could be largely because the diagnostic criteria are unclear and that the diagnostic equipment available is expensive. Handelsman et al. [15] proposed possible components of a vestibulotoxicity monitoring program comprising the head impulse test (HIT), dynamic visual acuity (DVA), postural control, head shake test, videonystagmography with caloric test, rotational test, video head impulse test (vHIT), vestibular evoked myogenic potentials (VEMPs), and posturography test. However, further study is needed to determine an ideal protocol for assessing vestibular function during and after ototoxic medication.

Systemic administration of ototoxic medication, including cisplatin chemotherapy, should reasonably affect both ears in the same way. It is also sensible to assume that the clinical manifestations of patients with cisplatin vestibulotoxicity would be similar to those who have suffered bilateral vestibulopathy from other causes, and the diagnostic principles of bilateral vestibulopathy could also be applied in this regard. Important diagnostic criteria of bilateral vestibulopathy by the Classification Committee of the Barany Society consist of chronic vestibular symptoms (unsteadiness plus either oscillopsia, or worsening of unsteadiness in darkness and/or on uneven ground), and bilaterally reduced vestibulo-ocular reflex (VOR) function documented by vHIT and/or caloric test and/or rotational test [16]. Nonetheless, there are also a wide range of related symptoms of drug-induced vestibulotoxicity evident in the published literature including dizziness, vertigo, nausea, and ataxia [17–19]. Valuable bedside examinations in the diagnosis of drug-induced vestibulotoxicity, recommended in the literature, are the HIT, DVA, and clinical test of sensory interaction of balance (CTSIB) [20].

Previous work investigating clinician knowledge, attitude, or practice regarding ototoxicity is sparse, and none particularly studied vestibulotoxicity. Steffens et al. [21] explored knowledge and attitude regarding cisplatin ototoxicity, and found that audiologists and oncologists had comprehensive knowledge and understanding of ototoxicity. The majority of respondents in that study thought that it was unlikely or slightly likely that balance disturbance would develop after cisplatin treatment. Studies exploring current practice suggested that ototoxic monitoring programs do not seem to be consistently implemented and the protocols vary across clinical settings [21, 22]. Cancer patients are not routinely asked about ototoxic effects in an oncology consultation [21, 23]. Typically, in the United Kingdom (UK), patients who complain of audiovestibular symptoms are referred either to Otolaryngology or Audiology Department. However, only 10% of audiovestibular professionals in the UK reported that balance assessment was part of ototoxicity monitoring in their centres [22].

This international study explored the knowledge, experiences, and opinions of audiovestibular healthcare professionals towards cisplatin vestibulotoxicity. The first objective addressed knowledge of drug-induced vestibulotoxicity, particularly on symptoms, clinical examination, and vestibular function tests. The second objective determined their experiences and whether they had come across any patients with cisplatin vestibulotoxicity. Last, the third objective sought opinions on the possibility of cisplatin vestibulotoxicity and the potential for a screening protocol.

## Methods

Ethical approval was obtained from the East Midlands—Nottingham Research Ethics Committee (18/EM/0369 date 30/04/2019). The approval of the online survey study was a part of a larger clinical study conducting to explore prevalence and impacts of vestibulotoxicity associated with cisplatin in adult survivors of cancer.

### Study sample

The target study population was healthcare professionals working in the audiovestibular field namely Audiologists, Audiovestibular Physicians, Otolaryngologists, and Vestibular Physiotherapists. To reach relevant participants, professional groups and social media channels were identified. An example of professional groups was the Balance Interest Group of the British Society of Audiology which is a multi-disciplinary team of professionals interested in balance disorders. The leaders of audiovestibular academic groups were contacted and asked to disseminate the invitation email with the survey link to all members of their group. Audiology and Otolaryngologist Facebook groups included Audiology-Vestibular science forum, Audiovestibular Medicine and Neuro-otology Interest Group, British Academy of Audiology, and American Academy of Audiology. The same information was posted on these social media groups by the member of the study team.

### Questionnaire development

A questionnaire (Appendix 1) was developed to answer the specific research questions through discussion and consensus within the study team. The questionnaire consisted of 13 questions: demographic data (4), knowledge (5), experience (2), and opinions (2). All of the questions were closed except one question asking about the geographical location. Response scales included single-choice option, multiple-choice options, and Likert scales. Questions assessing knowledge of clinicians regarding detection of drug-induced vestibululotoxicity were designed for the participants to choose more than one options if they know the answers, or they could choose to answer that they do not know. At the end of the questionnaire, there was an optional free text section asking for any comments and feedbacks which guided qualitative data for analysis. The survey was created through the online platform developed by the University of Nottingham. All respondents provided online consent and their participation was voluntary. To minimize missing data, and hence bias, the survey was programmed to require an answer for all questions before being able to proceed to the next set of questions. Questions were also customized to each respondent by a skip logic such that Q 10.1–10.4 were asked only if the response to Q 10 was “yes”.

### Piloting

As a pilot, five audiovestibular colleagues of the study team undertook the survey and feedback from these participants led to some minor changes. Some question clarifications were modified such as the phrase “bedside clinical examinations” was used instead of “clinical tests” to state that the question referred to physical examination at the time and place of patient care but not the objective tests. The example of adding more option in certain questions was to put “partial recovery” as a response option for the questions asking for outcome of the treated patient.

### Data analysis

The categorical data for all closed questions were descriptively analyzed, and data were expressed as percentages. Qualitative data from the final optional question were managed by content analysis process [24]. The initial step was to read and re-read the free text provided to get a sense of the overall comments. Then, the texts were dividing up into smaller meaningful parts, condensed meaning units. Each condensed meaning unit was further coded and collated. Codes were then organized into a category that text’s content belongs together. Then both quantitative and qualitative information were utilized to complement each other.

## Results

### Characteristics of participants

There were 93 responses from audiovestibular medical professionals. The characteristics of respondents are summarized in Table 1. Sixty-three percent of participants were Audiologists, 20% were Otolaryngologists, and 9% were Audiovestibular physicians. Fifty-nine percent of them were in senior level, and 31% were in mid-career position. Almost 60% had been working in their job roles for more than 10 years. Eighty-three percent of them worked in the UK. The following results are presented in accordance with the three research objectives.

**Table 1:** Characteristics of participants

Characteristic	Number (%)
Occupation	
Audiologists	59 (63%)
Otolaryngologists	19 (20%)
Audiovestibular physicians	8 (9%)
Vestibular Physiotherapist	4 (4%)
Others: Hearing Therapist 1, Advanced Practitioner 1, and Clinical Scientist 1	3 (3%)
Level of occupation	
Junior/trainee	9 (10%)
Mid-career	29 (31%)
Senior	55 (59%)
Year of working in the job role	
< 5 years	20 (22%)
5–10 years	18 (19%)
10–20 years	29 (31%)
> 20 years	26 (28%)
Country of the service	
United Kingdom	78 (84%)
Other countries: USA 2, Australia 2, India 2, Canada 1, South Africa 1, Portugal 1, Libya 1, Colombia 1, Denmark 1, Saudi Arabia 1, Egypt 1, Nepal 1	15 (16%)

### Knowledge

**Fig. 1:**
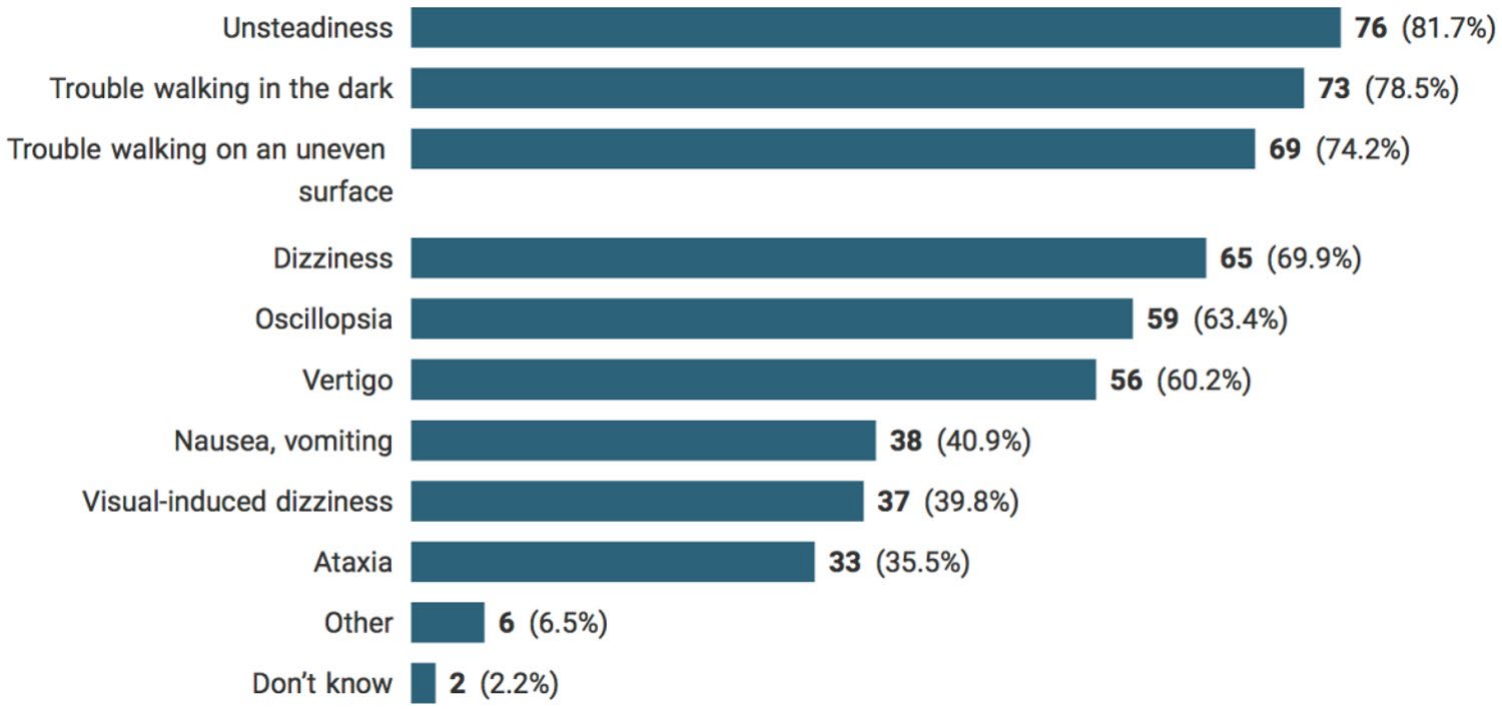
Indicative symptoms of drug-induced vestibulotoxicity

The most commonly selected answers of indicative symptoms of drug-induced vestibulotoxicity symptoms were unsteadiness (81.7%), trouble walking in the dark (78.5%), trouble walking on an uneven surface (74.2%), dizziness (69.9%), oscillopsia (63.4%), and vertigo (60.2%), nausea and vomiting (40.9%), visual-induced dizziness (39.8%), and ataxia (35.5%). Six percent of respondents reported other symptoms such as spatial disorientation, vague symptoms, features of endolymphatic hydrops, and head movement provoked vestibular symptoms. The average number of symptoms selected per participant was 5.9 (SD = 2.5). Only 2% of respondents stated that they did not know the symptoms of drug-induced vestibulotoxicity. Figure 1 reveals the participants’ responses concerning indicative symptoms of drug-induced vestibulotoxicity.

**Fig. 2:**
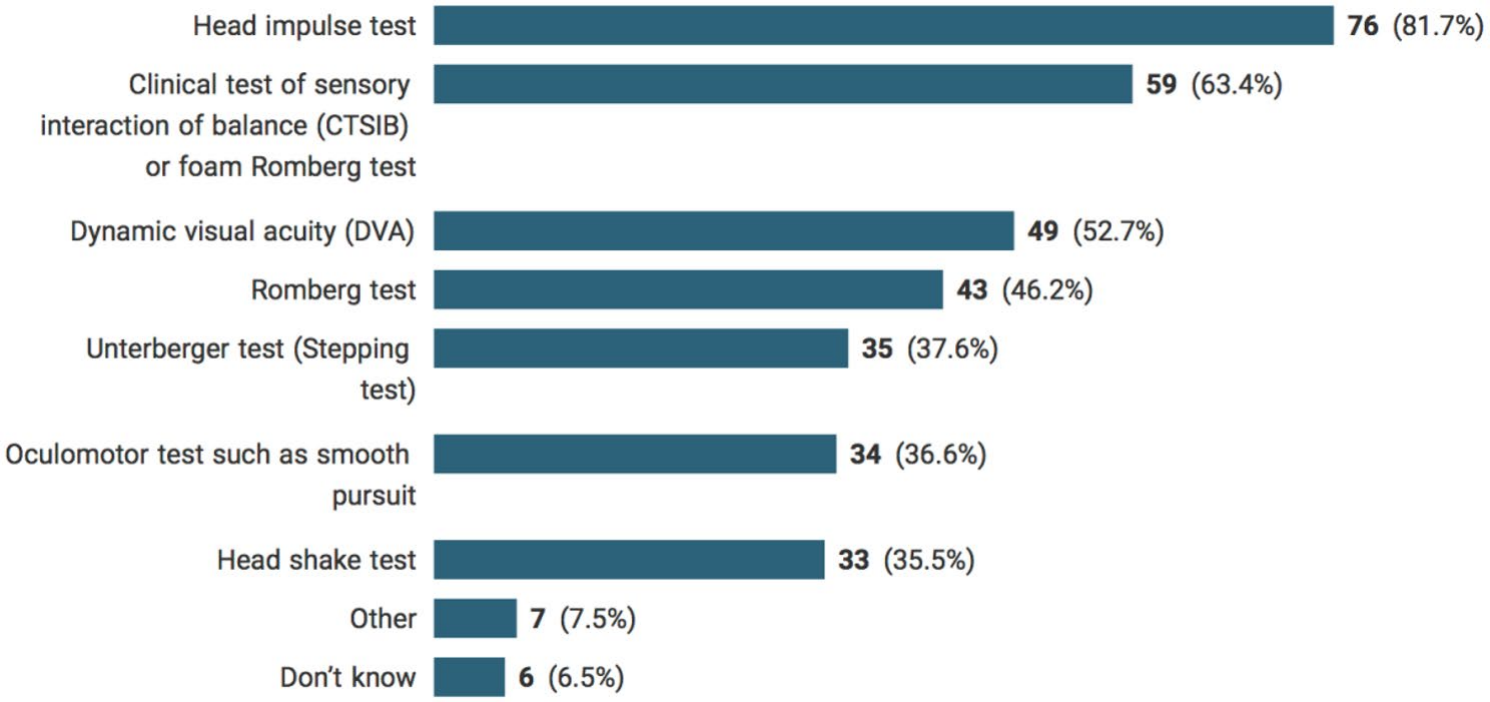
Useful physical examinations in detecting drug-induced vestibulotoxicity

Participants’ answers considering physical examinations of clinical benefit in detecting drug-induced vestibulotoxicity are displayed in Fig. 2. Physical examinations reported were the head impulse test (HIT) (81.7%), clinical test of sensory interaction of balance (CTSIB) (63.4%), and dynamic visual acuity (DVA) (52.7%), followed by Romberg test, Unterberger test (stepping test), oculomotor test, and head shake test. Examples of other physical examinations were full neurological examination and positional tests. The average number of symptoms selected per participant was 3.7 (SD = 1.9). Six percent of participants reported they did not know what to answer.

**Fig. 3:**
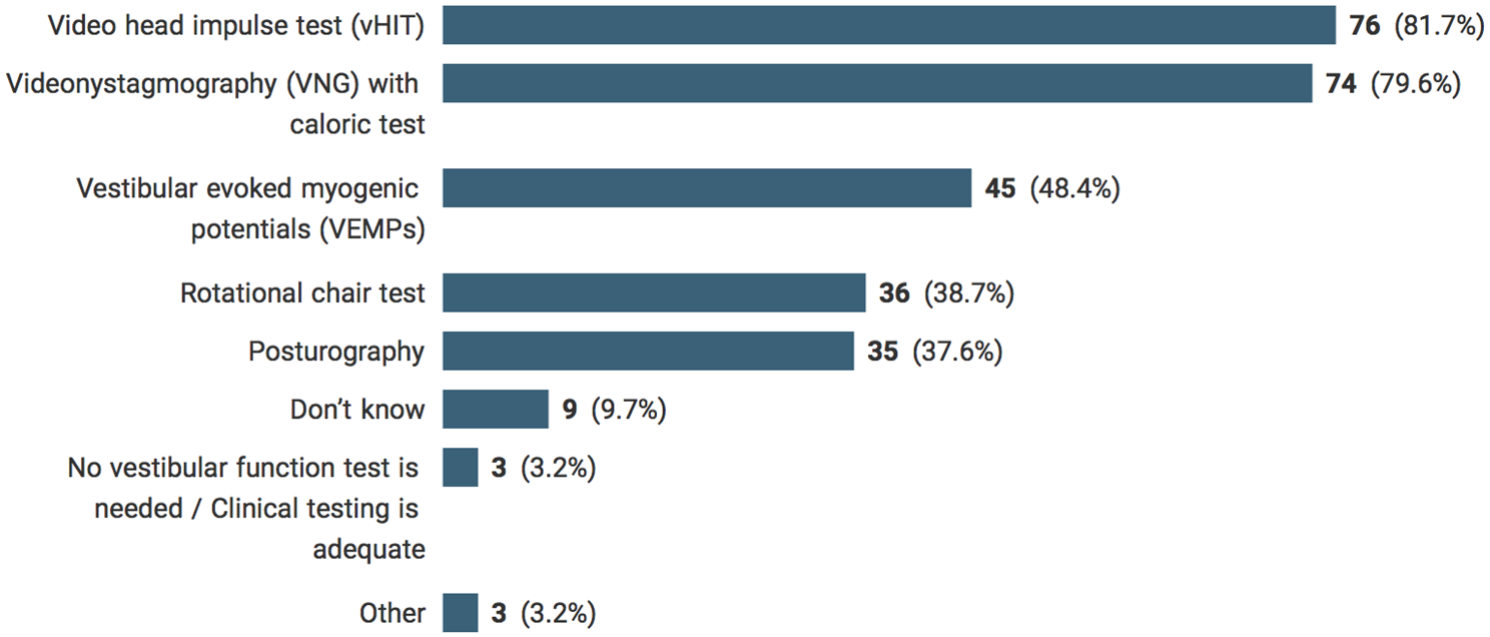
Useful vestibular function tests in detecting drug-induced vestibulotoxicity

Vestibular function tests of clinical value in detecting drug-induced vestibulotoxicity reported by participants were video head impulse test (vHIT) (81.7%), videonystagmography with caloric test (79.6%), vestibular evoked myogenic potentials (VEMPs) (48.4%), rotational chair test (38.7%), and posturography (37.6%). Three percent indicated that no vestibular function test was needed and clinical testing was sufficient for the diagnosis of drug-induced vestibulotoxicity. An example of another test was the suppression head impulse test (SHIMP), a new paradigm of vHIT [25]. Nearly 10% of participants indicated that they did not know what to answer. Figure 3 shows the participants’ responses reflecting vestibular function tests of clinical value.

Regarding confidence levels of participants in diagnosis and management of drug-induced vestibulotoxicity, approximately 40% of respondents reported that they were confident. Another 30% reported that they were neither confident nor unconfident. Almost 30% of them reported they were either somewhat unconfident or very unconfident. There was a high association between the degree of confidence in diagnosis and in management, demonstrated by Spearman’s rank correlation value of 0.79.

### Experiences

Thirty-one (33%) respondents reported consultations with patients with cisplatin vestibulotoxicity. Thirty-eight percent reported that they had never seen any patients with this condition, and 29% of participants were not sure about this.

Participants’ experiences of the condition were associated with the degree of confidence in diagnosis and management. Seventy-seven percent of respondents who had seen patients with cisplatin vestibulotoxicity felt confident in making diagnosis of drug-induced vestibulotoxicity, compared to 31% and 22% in the group with no experience and the unsure group, respectively. Furthermore, 37% of participants in the no experience and unsure groups were unconfident in making the diagnosis. Sixty-five percent of respondents who had seen patients were confident in the management of the condition, whilst there were 29% in no experience group and 22% in the unsure group. Thirty-four percent of participants in the no experience and unsure groups felt unconfident in the management.

**Fig. 4:**
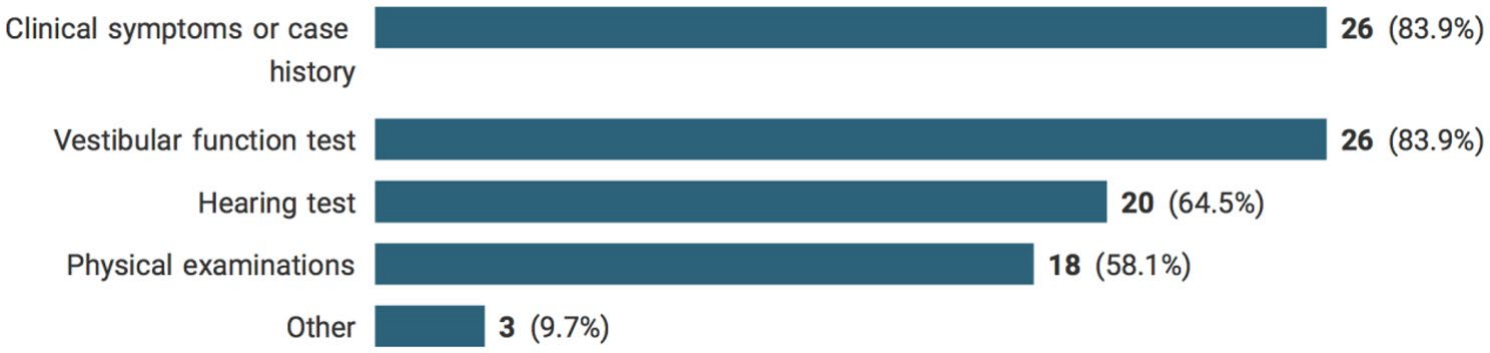
Criteria for diagnosis patient with cisplatin vestibulotoxicity

Participants who reported that they had seen patients with the condition were further asked about their experiences. Clinical symptoms and vestibular function tests were parts of diagnostic criteria, described by more than 80% of respondents. Physical examinations and hearing test were also utilized by approximately 60%. Others were based on neurological examinations, or the diagnosis made by other clinicians. A report of diagnostic components for patients with cisplatin vestibulotoxicity is showed in Fig. 4.

Clinical experience of clinicians to cisplatin vestibulotoxicity is summarized in Table 2. Nineteen of the 31 (61.3%) participants who had seen patients with cisplatin vestibulotoxicity reported that they had seen 2–5 patients with the condition. Eight participants (25.8%) indicated that they had seen more than five patients, whilst four participants (12.9%) reported that they had seen one patient with the condition. Twenty-nine participants (93.5%) treated the patients by vestibular rehabilitation. Some provided counselling (51.6%), and some referred patients to other specialties for management (16.1%). Two clinicians (6.5%) stated that discussions were held with oncology colleagues to consider adjusting the cisplatin dose or seeking alternative medication. Only one participant (3.2%) treated the patient with medication. Partial recovery was the most common outcome of the management that was reported by 23 participants (74.2%), followed by persistent symptoms reported by four participants (12.9%). Only one clinician (3.2%) stated that his/her patient(s) had complete recovery. Three participants (9.7%) were unsure about the outcomes as the patients were followed up outside their services.

**Table 2:** Clinician experience with cisplatin vestibulotoxicity

Clinician experience	Number (%)
Number of patients seen	
1	4 (12.9%)
2–5	19 (61.3%)
> 5	8 (25.8%)
Management	
Vestibular rehabilitation	29 (93.5%)
Counselling	16 (51.6%)
Referral to other specialties for management	5 (16.1%)
Medication	1 (3.2%)
Other: Decisions taken at oncology department	2 (6.5%)
Outcome	
Complete recovery	1 (3.2%)
Partial recovery	23 (74.2%)
Persistent symptoms	4 (12.9%)
Other: Unknown	3 (9.7%)

Fifty-five (59.1%) participants had seen and managed patients with drug-induced vestibulotoxicity caused by other medications, which were aminoglycoside antibiotics (94.5%), loop diuretics (12.7%), and nonsteroidal anti-inflammatory drugs (7.3%).

### Opinions on the possibility of cisplatin vestibulotoxicity and a screening protocol

Thirty-two percent of respondents thought that cisplatin often causes vestibulotoxicity, and 52% of them thought that cisplatin vestibulotoxicity is possible. Three percent thought that cisplatin always causes vestibulotoxicity, and 4% thought the effect was unlikely. Nine percent answered that they do not know, and none of them answered that cisplatin cannot cause vestibulotoxicity.

**Fig. 5:**
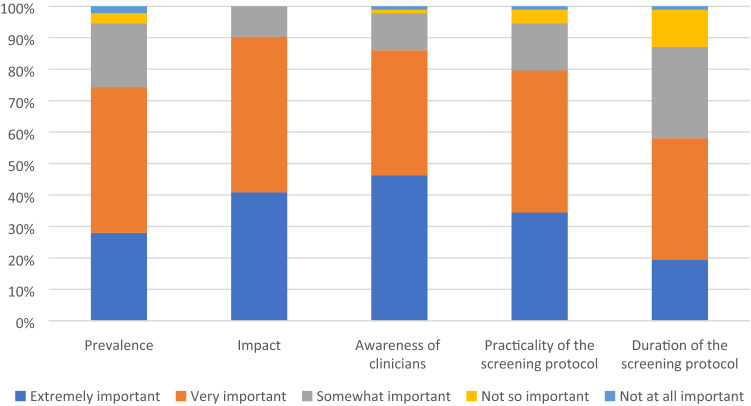
Opinions on factors contributing to the desirability of a vestibulotoxicity screening protocol

Figure 5 displays respondents’ opinions of the importance of each factor on the desirability of a vestibulotoxicity screening protocol. More than 80% of participants acknowledged that the impact of vestibulotoxicity, awareness of clinicians, and practicality of the vestibular screening protocol were extremely or very important. The prevalence of vestibulotoxicity and duration of the screening protocol were thought to be extremely or very important by 70% and 60% of participants, correspondingly.

### General comments

Twenty-five statements were provided in the free text option. These were coded and categorized into four themes: knowledge (four comments), experience (seven comments), opinions on screening protocol (ten comments), and current practice (four comments). Explanations and examples are given below.

#### Knowledge

Some comments emphasized the importance of understanding the effect of ototoxic medication on cochlear and vestibular functions, which will further lead to increased awareness of ototoxic drug and its impact. An example of these statements was “Clinicians should notice the ototoxic agents and the degree of disability they cause”. One respondent stated that “I now feel totally incompetent. So now I shall read up and learn”, suggesting the self-perceived low confidence in dealing with the patient whilst willing to learn more. Not only sufficient knowledge on the topic but also practical clinical skill seems to be crucial in clinical practice, for example, “Interpreting head impulse testing takes some experience and expertise”.

#### Experience

The participants reported varying degree of exposure to vestibulotoxicity. One respondent stated that “Cisplatin seems to be more cochleotoxic than vestibulotoxic”, this was also confirmed further by another respondent specified that “I always inform about the possibility but I only see very few cases”. In addition, it is difficult to disentangle vestibulotoxic effect between general deconditioning of cancer patients receiving cisplatin. Vestibular rehabilitation seems to be important in relieving patient symptoms and better quality of life. One respondent commented that “If caught early then rehabilitation can be put in place sooner. The sooner someone is seen for rehabilitation the better especially when looking at the anxiety that imbalance can cause”.

#### Opinions on a screening protocol

Clinical judgement appears to be influential in opinions on a screening protocol, for example, one participant commented that “I think screening with DVA would be an appropriate bedside test, and vHIT for the lab test. Rotatory chair is more sensitive, but unfit as a screening tool”. Clinical experience also plays a significant role. One example of these statements was “In our experience, benign paroxysmal positional vertigo could occur secondary to damage caused by vestibulotoxic medication and should be looked for in screening protocol”.

#### Current practice

Current practice regarding vestibular screening program has been reported to be varying from no testing at all to full examination in all patients receiving cisplatin. One respondent emphasized that “We perform screening pure tone audiogram before commencing ototoxic medications for patients receiving treatment for testicular cancer but not vestibular screening”, indicating the implementation of only hearing surveillance protocol. Whilst another participant mentioned that “In my hospital, vestibular examination is integral to manage patients receiving cisplatin and other compounds”. One respondent also stated that “I screen using the rotating chair in a paediatric service”.

## Discussion

This survey received a response from relevant healthcare professionals including 60% being ‘senior’ clinicians. Some general comments from the respondents could confirm and explain the quantitative results of the survey. The discussion gathers the insights together in summary form and makes some comments on the findings.

### Diagnosis of the cisplatin vestibulotoxicity

The common selected answers of indicative symptoms, bedside examinations, and vestibular function test of drug-induced vestibulotoxicity were most closely associated with the proposed diagnostic criteria of vestibulotoxicity and bilateral vestibulopathy [15, 16, 20]. It is also recognized by healthcare professionals that there were a wide range of possible suggestive symptoms of drug-induced vestibulotoxicity, which is congruent with the literature [17–19]. The majority of respondents agreed that some kind of quantitative vestibular function test was needed, and bedside testing alone was not sufficient to make the diagnosis of cisplatin vestibulotoxicity.

Most clinicians stated the utility and usefulness of vHIT in diagnosing drug-induced vestibulotoxicity, possibly because its practicality, portability, and high specificity in the diagnosis of vestibular disorders [26]. So far, only one study evaluated VOR function using vHIT and revealed 3 out of 12 (25%) paediatric cancer patients had decreased VOR gain [27]. Interestingly, rotational chair testing, which is considered to be the gold standard in diagnosing bilateral vestibular loss [28, 29], was reported to be useful in the diagnosis by a substantially fewer number of respondents compared to that of the vHIT and caloric test. A qualitative statement mentioned that the rotatory chair is unfit as a screening tool though its high sensitivity could be one of the explanations of this finding. Nearly half of participants reported the usefulness of VEMPs in making the diagnosis. VEMPs is one possible component in a proposed vestibulotoxicity monitoring program [15], but it is not included in the diagnostic criteria of bilateral vestibulopathy [18]. This could be because the degree of otolith dysfunction appears to be less than that of canal dysfunction in bilateral vestibulopathy [16]. None of the studies evaluated otolith function by VEMP in cancer populations who received cisplatin chemotherapy [12]; therefore, its utility has not yet been fully investigated.

### Clinical experience

It is well-known that aminoglycoside antibiotics and cisplatin chemotherapy are the most common medications causing ototoxic effects [7]. Thirty-three percent of the respondents reported that they had come across patients with cisplatin vestibulotoxicity, whilst 56% had seen patients with vestibulotoxicity caused by aminoglycoside antibiotics. The results suggest that vestibulotoxicity associated with cisplatin chemotherapy may not be as uncommon as it seemed in the literature. In this study, most of the respondents thought that cisplatin often or possibly causes vestibulotoxicity. On the other hand, in another study, the likelihood of developing balance disturbances in patients receiving cisplatin was reported to be “unlikely” to “slightly likely” by the majority of audiologists and oncologists [21]. This could be because the opinions were gathered from different clinical settings.

Confidence level in diagnosis and management of the condition was corresponding to the clinical experience whether they had seen any patient or not. Remarkably, one-third of them reported being unsure whether they have come across this condition or not. The reasons behind this were not available from the survey results and should be further investigated. These findings show uncertainty in making the diagnosis and management of drug-induced vestibulotoxicity, indicating that there is substantial room for improvement.

The outcomes of patients with cisplatin vestibulotoxicity were mostly described to be poor, even after vestibular rehabilitation, which corresponds with the existing literature that the prognosis of bilateral vestibular hypofunction is poor and most patients do not improve with time [30]. Consequently, early identification of a patient at risk or prompt diagnosis could help prevent permanent debilitating balance problems.

### Suggestions for clinical practice

We suggest some practical strategies to improve the quality of management pathway of cisplatin and other drug-induced vestibulotoxicity. First, more training at individual level should be encouraged. Although a lot of respondents had never dealt with any patients hence did not have direct experience, a sufficient level of knowledge to detect the condition with a high index of suspicion should be stimulated. The provision of lectures, workshops, and case discussions could be beneficial in this regard. Second, clinical consultation with more experienced specialists should be supported in each clinical practice. Direct clinical experience had significant influence on confidence level in diagnosis and management; hence, educational role of more experienced professionals should be promoted.

Currently, vestibulotoxicity monitoring program is not regularly undertaken in clinical settings [22]. A flexible and compassionate approach is necessary especially when quantitative vestibular function test is not available or transportation of patients to laboratory setting is not possible. Bedside testing is certainly preferable to no testing at all [31].

Although most audiovestibular healthcare professionals in this study were aware of potential cisplatin vestibulotoxicity, it is the Oncologists who will be the clinicians that will come across the affected patient initially. Therefore, working in collaboration with Oncologists is crucial in this situation. The present study did not determine levels of awareness amongst Oncology clinicians. A recent study emphasized the role of the Audiologist as a clinical team member in the care plan of patients receiving ototoxic medication [32]. The management of ototoxicity ideally should be based on a team approach involving both Audiologists and Oncologists [21].

## Limitations

There are some limitations to be taken into account when interpreting the results of this study. First, participants were self-enrolled into the study which could lead to selection bias. For example, it can be assumed that clinicians were more likely to have undertaken the survey if they had knowledge of cisplatin vestibulotoxicity or were interested in the topic. It is also possible that clinicians did not want to admit that they did not previously know about cisplatin vestibulotoxicity and therefore might exaggerate their current knowledge and experience in the subject. This could occur as many items in the questionnaire had potentially leading questions. However, we believe that the results are reasonably illustrative view of audiovestibular professionals since we approached potential participants via relevant academic and social media groups who seem to be working actively in clinical settings. Second, convenience sampling limits the interpretation of results to the specific context including population group, clinical setting, and country of the service. For example, most of respondents in this study are working in the UK so the findings might represent UK perspectives and contexts. The findings still provide a snapshot of the current situation and practice.

## Conclusion

Most of the audiovestibular professionals in this survey are aware of potential vestibulotoxicity associated with cisplatin chemotherapy, and some had seen patients with the condition. However, there is substantial room for improvement in knowledge, diagnostic protocol, and management. Relevant healthcare professionals should refresh their knowledge and actively promote their roles in the diagnosis and management of vestibulotoxicity. In summary, this study provides a better understanding of the perspectives of audiovestibular clinicians of cisplatin vestibulotoxicity, which will underpin appropriate detection and management of this debilitating condition.

## Appendix 1: Questionnaire

1. What is your occupation?

- Audiologist
- Audiovestibular physician
- ENT doctor
- Vestibular physiotherapist
- Other (please specify)

2. What level of your occupation would you say you were?

- Student
- Junior/traine
- Mid-career
- Senior

3. How long have you been working in your job role?

- < 5 years
- 5–10 years
- 10–20 years
- > 20 years

4. Which country is the location of your service?

5. What do you think are the indicative symptoms of patients who have drug-induced vestibulotoxicity?—You may choose more than one option.

- Vertigo
- Dizziness
- Unsteadiness
- Ataxia
- Oscillopsia
- Trouble walking in the dark
- Trouble walking on an uneven surface
- Visual-induced dizziness
- Nausea, vomiting
- Don’t know
- Other (please specify)

6. What bedside clinical examinations do you think are useful to detect patients who may have drug-induced vestibulotoxicity?—You may choose more than one option.

- Oculomotor test such as smooth pursuit
- Head impulse test
- Head shake test
- Dynamic visual acuity (DVA)
- Romberg test
- Clinical test of sensory interaction of balance (CTSIB) or foam Romberg test
- Unterberger test (stepping test)
- Don’t know
- Other (please specify)

7. What vestibular function tests do you think are useful to detect patients who may have drug-induced vestibulotoxicity?—You may choose more than one option.

- No vestibular function test is needed/clinical testing is adequate
- Videonystagmography (VNG) with caloric test
- Rotational chair test
- Video head impulse test (vHIT)
- Vestibular evoked myogenic potentials (VEMPs)
- Posturography
- Don’t know
- Other (please specify)

8. How confident are you in the diagnosis of drug-induced vestibulotoxicity?

- Confident
- Neither confident nor unconfident
- Unconfident

9. How confident are you in the management of drug-induced vestibulotoxicity?

- Confident
- Neither confident nor unconfident
- Unconfident

10. Have you ever come across patient(s) who has vestibulotoxicity associated with cisplatin?

- Yes. If Yes, then answer Q10.1–10.4
- No. If No, then answer Q11
- Not sure. If Not sure, then answer Q11

10.1 How did you diagnose patient with this condition?—You may choose more than one option.

- Clinical symptoms
- Physical examinations
- Vestibular function test
- Hearing test
- Other (please specify)

10.2 How many patients with presumed cisplatin vestibulotoxicity have you seen in total?

- 1
- 2–5
-  > 5

10.3 How did you treat them?—You may choose more than one option.

- Counselling
- Referred to other specialty for proper diagnosis
- Referred to other specialty for proper management
- Medication
- Vestibular rehabilitation
- I did not know how to treat them
- Other (please specify)

10.4 What was the outcome of (most of) the treated patient with cisplatin vestibulotoxicity?

- Complete recovery
- Partial recovery
- Persistent symptoms
- Other (please specify)

11. Have you ever seen and managed patients with other drug-induced causes of vestibulotoxicity?

- Yes. If Yes, what was the drug?—You may choose more than one option
- Aminoglycoside antibiotics
- Loop diuretics
- NSAIDs
- Other (please specify)
- No
- Not sure

12. To what extent do you think that cisplatin can cause vestibulotoxicity in adults?

- Yes, always
- Yes, often
- Yes, possible
- Yes, but unlikely
- No
- Don’t know

13. How important do you think the following factors are in contributing to your perspective on the desirability of a vestibulotoxicity screening protocol? (Please select one answer in each row.)

**Table:**

	Extremely important	Very important	Somewhat important	Not so important	Not at all important
Prevalence of vestibulotoxicity					
Impact of vestibulotoxicity					
Awareness of clinicians					
Practicality of the vestibular screening test					
Duration of the vestibular screening assessment					

14. General comments.
